# Implementation of a clinical nursing pathway for percutaneous coronary intervention: A randomized controlled trial protocol

**DOI:** 10.1097/MD.0000000000022866

**Published:** 2020-10-23

**Authors:** Zhimin Zhang, Jincheng Bai, Yongmei Huang, Lingling Wang

**Affiliations:** a Department of Cardiovascular Medicine; b Department of Nursing; c Department of Radiology, Changshan County People's Hospital, Zhejiang, China.

**Keywords:** clinical nursing pathway, percutaneous coronary intervention, protocol

## Abstract

**Background:**

Acute myocardial infarction is a very common disease in the emergency room. Emergency percutaneous coronary intervention (PCI) is the first choice to open infarct-related artery in time to regain the active blood flow of myocardial tissue. Clinical nursing pathway (CNP), namely clinical project, is an original nursing mode with good quality, outstanding efficiency, and low treatment spending, so it has attracted more and more attention. However, few studies have reported the implementation of a CNP in PCIs. The purpose of the protocol is to assess the impact of CNP on the clinical efficacy of transradial emergency PCI.

**Methods:**

This is a randomized controlled, single center trial which will be implemented from January 2021 to June 2021. Hundred samples diagnosed with acute myocardial infarction will be included in this study. It was authorized via the Ethics Committee of Changshan County People's Hospital (CCPH002348). Patients are assigned to the following groups: control group, given normal routine care; CNP group, treated with CNP plan. The time from door to balloon, hospitalization expenses, length of stay, postoperative complications, patients’ satisfaction with treatment are compared and analyzed. All data are collected and analyzed by Social Sciences software version 21.0 (SPSS, Inc., Chicago, IL) program.

**Results:**

Differences of clinical outcomes between groups (Table 1).

**Conclusion:**

This original evidence-based nursing model can be used as the foundation for further research.

**Trial registration number:**

researchregistry6030

## 1 Introduction

Cardiovascular disease (CVD) is one of the main causes of human death.^[1]^ According to the 2015 Global Burden of Disease Research, there were 427.7 million CVD cases and 17.92 million CVD-related deaths in 2015. It has been predicted that the number of deaths due to heart disease will reach 23.3 million by 2030.^[2]^ Approximately 50% of mortality related to CVD is caused by coronary atherosclerotic heart disease.^[3]^ Patients with severe or instable coronary heart disease need percutaneous coronary intervention (PCI).^[4,5]^ Current treatment options suggest that patients with acute ST segment elevation myocardial infarction should be dealt with PCI.^[6,7]^ With the increase of life expectancy, there will be more and more elderly patients with coronary heart disease.

The risks related to PCIs differ greatly, and special care is needed in independent and collaborative settings. Risk elements such as age, anticoagulation therapy, repeated PCI, gender, and arterial access management have a great influence on consequences.^[8–10]^ Medical management and secondary prevention strategies for PCI are procurable from international organizations. The role of nurses and the influence of nursing interventions on health consequences are rarely described and analyzed in these guidelines. At present, with the rapid progress of medical technology, the nursing mode must be updated accordingly. Clinical nursing pathway (CNP), namely clinical project, is an original nursing mode with good quality, outstanding efficiency, and low treatment spending, so it has attracted more and more attention.^[11,12]^ However, few studies have reported the implementation of a CNP in PCIs. The purpose of the protocol is to assess the impact of CNP on the clinical efficacy of transradial emergency PCI.

## 2 Materials and methods

### 2.1 Study design

This is a randomized controlled, single-center trial which will be implemented from January 2021 to June 2021. This trial is conducted according to the SPIRIT Checklist of randomized researches. It was allowed by the Ethics Committee of Changshan County People's Hospital (CCPH002348), and it has been registered in the research registry (researchregistry6030).

### 2.2 Subjects

Hundred patients with diagnosed acute myocardial infarction (AMI) will be included in this experiment. In the random envelope, a random number is assigned to whole patients through the random-number table, and the distribution result is invisible. Patients are assigned randomly to the following groups:

(1) control group, accepting normal nursing plan, containing 50 patients;
(2) CNP group, treated with CNP plan.

Patient inclusion criteria contains:

(1) acute ST segment elevation myocardial infarction;
(2) emergency transradial PCI therapy is necessary.

Patient exclusive criteria contains:

(1) Damage to organs such as kidney, lung, and so on;
(2) Malignant tumor;
(3) a history of coagulopathy, a history of the history of renal and hepatic dysfunction.

Ensure that informed consent forms signed by all participants have been collected.

### 2.3 Nursing protocol

The control group is given normal nursing before and after hospitalization. In order to estimate the development of the patient's condition, emergency electrocardiogram examination is performed during hospitalization. To guard against complications, including multiple procedures, such as preoperative skin preparation, constant monitoring of oxygen, vital signs, postoperative injury care, and so on. Under the guidance of doctors, nurses give patients medication and preoperative preparation. CNP group adopted clinical nursing method. The patients is dealt with rapid diagnosis, then there is a more precise examination, equipment of vital signs support and emergency drugs, and quickly transferred to the correlative experts. At the date of hospitalized, the nursing group formulates the corresponding nursing plan according to the current situation of the patients, referring to the domestic and foreign standards, and modified the nursing content on the basis of the matters met in the actual operation. The patients are instructed to complete medication and nursing plan. The nursing group assesses the patient's general condition and uses emergency equipment and appropriate drugs to prevent adverse events. When the patient is discharged from the hospital, he will receive a pamphlet for AMI, introducing healthy lifestyle, drug treatment, and arranging follow-up.

### 2.4 Outcomes

Among the 2 groups, the time from door to balloon, hospitalization expenses, length of stay, postoperative complications, patient's satisfaction with treatment are compared and analyzed.

### 2.5 Statistical analysis

All data are collected and analyzed by Social Sciences software version 21.0 (SPSS, Inc., Chicago, IL) program. The categorical variables are expressed as percentage of patients and analyzed by the Fisher exact test or Pearson Chi-square test. Continuous variables are showed as mean (standard deviation) and analyzed by paired *t* tests or independent *t* tests. The *P*-value of significance level is set to be less than .05.

## 3 Results

Comparison of clinical outcomes between groups (Table 1)

**Table 1:**
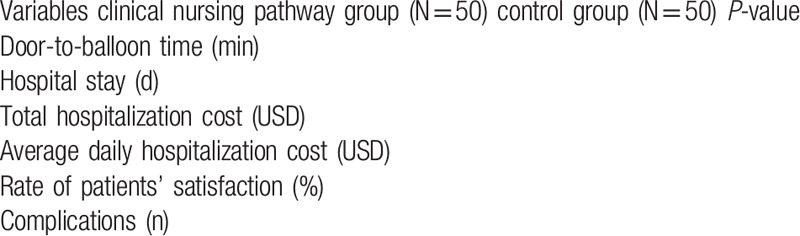
Comparison of clinical outcomes between groups.

## 4 Discussion

AMI is a very common disease in the emergency room.^[13,14]^ The primary long-term aim of AMI treatment is not only to make patients survive at the onset of the disease, but also to retain myocardial reserve as far as possible to maintain the level of life after infarction.^[15,16]^ Emergency PCI is the first choice to open infarct related artery to renovate the active blood flow of damaged myocardial tissue. This method has been widely applied in the recovery of reperfusion in AMI.^[17,18]^ PCI within 4 to 6 hours after AMI is the most prosperous method to preserve infarcted myocardium.^[19]^ Thus, reducing door to balloon time is essential to improve short-term and long-term survival rates.^[20]^

CNP includes the supply of divinable and specific guidance for certain groups of patients included in the daily nursing scheme. This method fully embodies the functional characteristics of nursing as an occupation, and guides patients to carry out rehabilitation guidance, which is opportune, coordinated, and actual. CNP provides the most accurate and active nursing to attain the best clinical effect.^[21,22]^ We assume that compared with the traditional nursing mode, the operation of CNP in emergency PCI can enhance the quality of nursing, including dramatically shortening the time from door to balloon, reducing the length of hospital stay, reducing the total cost of hospitalization, reducing postoperative complications and increasing patients’ satisfaction.

## 5 Conclusion

This original evidence-based nursing model can be used as the foundation for further research.

## Author contributions

**Data curation:** Jincheng Bai.

**Investigation:** Yongmei Huang.

**Project administration:** Lingling Wang.

**Resources:** Lingling Wang.

**Software:** Lingling Wang.

**Writing – original draft:** Zhimin Zhang.

**Writing – review & editing:** Zhimin Zhang.
